# Autophagy regulation by acetylation—implications for neurodegenerative diseases

**DOI:** 10.1038/s12276-021-00556-4

**Published:** 2021-01-22

**Authors:** Sung Min Son, So Jung Park, Marian Fernandez-Estevez, David C. Rubinsztein

**Affiliations:** 1 Department of Medical Genetics, University of Cambridge, Cambridge, UK; 2 UK Dementia Research Institute, Cambridge Institute for Medical Research (CIMR), University of Cambridge, Cambridge, UK

**Keywords:** Acetylation, Alzheimer's disease

## Abstract

Posttranslational modifications of proteins, such as acetylation, are essential for the regulation of diverse physiological processes, including metabolism, development and aging. Autophagy is an evolutionarily conserved catabolic process that involves the highly regulated sequestration of intracytoplasmic contents in double-membrane vesicles called autophagosomes, which are subsequently degraded after fusing with lysosomes. The roles and mechanisms of acetylation in autophagy control have emerged only in the last few years. In this review, we describe key molecular mechanisms by which previously identified acetyltransferases and deacetylases regulate autophagy. We highlight how p300 acetyltransferase controls mTORC1 activity to regulate autophagy under starvation and refeeding conditions in many cell types. Finally, we discuss how altered acetylation may impact various neurodegenerative diseases in which many of the causative proteins are autophagy substrates. These studies highlight some of the complexities that may need to be considered by anyone aiming to perturb acetylation under these conditions.

## Neurodegeneration: Altered protein tagging impairs brain’s clean-up mechanisms

Modulation of a chemical tagging process known as acetylation could help brain cells remove toxic proteins associated with Alzheimer’s disease, amyotrophic lateral sclerosis (ALS) and other neurodegenerative disorders. David Rubinsztein and colleagues from the University of Cambridge, UK, review how enzymes that add or remove acetyl groups to or from proteins help to regulate autophagy, the process whereby damaged cellular components are removed. In neurons, imbalanced acetylation can result in the toxic build-up of proteins such as tau (Alzheimer’s disease), alpha-synuclein (Parkinson’s disease), huntingtin (Huntington’s disease) and superoxide dismutase 1 (ALS). The ensuing protein aggregation can then trigger progressive neuron loss and impaired function, especially as the brain ages. The authors propose that therapeutic targeting of acetylation to restore healthy autophagic activity could therefore have neuroprotective effects in many of these diseases.

## Introduction

Macroautophagy (hereafter autophagy) is a process mediating the delivery of cytoplasmic components to the lysosome for degradation via double-membrane vesicles called autophagosomes^1^. In mammalian cells, autophagosomes are formed from cup-shaped precursor structures called phagophores, which include a complex of autophagy proteins, including ATG5, ATG12 and ATG16L1^2^. The membranes of phagophores expand and form enclosed autophagosomes, and completed autophagosomes subsequently fuse with lysosomes^2,3^. Lysosomal digestion of autophagic cargoes protects cells against starvation and related stresses by releasing recycled building blocks from autophagic substrates.

Acetylation is a major posttranslational modification (PTM) and affects diverse aspects of protein function by altering properties such as stability, hydrophobicity, enzymatic activity, subcellular localization and interactions with other substrates and cofactors in the cell^4^. In acetylation, the acetyl group of an acetyl-coenzyme (Ac-CoA) can be co- or posttranslationally transferred to either the α-amino group of the N-terminus of a protein (Nt-acetylation) or to the ε-amino group of a lysine residue (K-acetylation). Nt-acetylation is catalyzed by highly conserved Nt-acetyltransferases (NATs) and is considered irreversible. On the other hand, K-acetylation is a reversible modification mediated by lysine acetyltransferases (KATs) at the ε-amino group of lysine residues. The tight regulation of acetylation by these enzymes plays fundamental regulatory roles in development and diverse human diseases, including diabetes and neurodegenerative conditions^4^.

In this review, we describe how autophagy is regulated by acetylation, particularly K-acetylation, by previously identified KATs and deacetylases (KDACs). We also summarize the therapeutic targeting of acetylation, which may potentially lead to effective strategies to treat neurodegenerative diseases.

## Introduction to autophagy

**Fig. 1:**
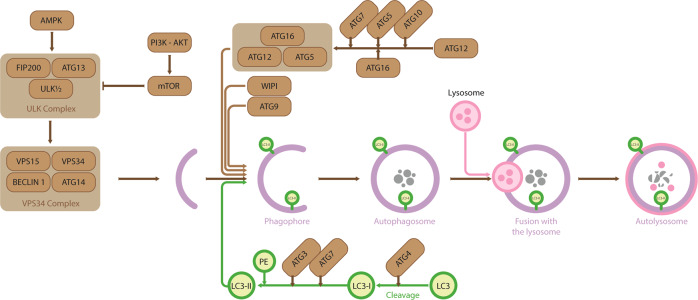
Overview of the autophagy pathway. mTORC1 inhibition and AMPK activation positively regulate the ULK1 complex through a series of phosphorylation events. The ULK1 complex subsequently activates VPS34/PI3K complexes, which leads to PI3P synthesis and the nucleation of preautophagosomal structures. PI3P then recruits PI3P effector proteins, namely, WIPIs and the ATG12-ATG5-ATG16L1 complex, which is essential for autophagosome membrane elongation. Subsequent fusion to lysosomes results in the degradation of a variety of substrates, such as protein aggregates, infectious agents, and damaged mitochondria.

Under normal conditions, cells sustain basal levels of autophagy to maintain homeostasis. However, a variety of stimuli, including nutrient deprivation, metabolic imbalance or cellular stress, can activate autophagy^2,3^. Autophagosome biogenesis includes three early stages: initiation, nucleation, and expansion of the isolation membrane (Fig. 1), and the process is mediated by autophagy-related proteins (ATGs)^2^, which were initially discovered in yeast^5^. Many ATG proteins can be regulated by PTMs, such as phosphorylation, ubiquitination and acetylation^6^. The Unc-51-like autophagy-activating kinase (ULK) 1/2 complex (consisting of ULK1, ATG13, RB1-inducible coiled-coil protein 1 (FIP200) and ATG101) plays a major role in autophagy as a signaling node for several pathways and by phosphorylating downstream effectors. During the initiation of autophagosome formation, this complex acts as a serine/threonine kinase that phosphorylates Beclin 1 in the vacuolar protein-sorting 34/PI3-kinase (VPS34)/PI3K complex^7^. Additionally, the ULK1 complex recruits ATG9, which is thought to be involved in delivering membranes to autophagosomal structures and may act as a lipid scramblase^8–10^. The VPS34/PI3K complex generates phosphatidylinositol 3-phosphate (PI3P), which facilitates the recruitment of WD-repeat protein-interacting with phosphoinositides (WIPI2) that recruits the ATG5-ATG12-ATG16L1 complex to the sites of phagophore formation^11^. This complex enables the conjugation of LC3 and its family members to phosphatidylethanolamine in phagophore membranes^12^. To degrade the autophagosomal content, autophagosomes must fuse with a functional lysosome, and SNARE proteins mediate this fusion^13^.

Autophagy is tightly regulated by intracellular and extracellular signals^14^. Mechanistic target of rapamycin (mTOR) complex 1 (mTORC1) integrates signals related to growth and metabolism in response to nutrient and energy levels and negatively regulates autophagy^15^ through the hosphorylation of ULK1, ATG13, transcription factor EB (TFEB) and other autophagy-related proteins under nutrient-rich conditions.

## Regulation of acetylation in cells

### Nt-acetylation

Six different NATs have been identified in mammals (NatA to NatF)^16^. NATs regulate the transfer of an acetyl group from Ac-CoA to the free α-amino group of a polypeptide chain that is being synthesized. NATs can differ in their subunit composition and substrate specificity^17^. Nt-acetylation can regulate the subcellular localization of proteins, protein stability, and protein-protein interactions^4^.

### K-acetylation

**Fig. 2:**
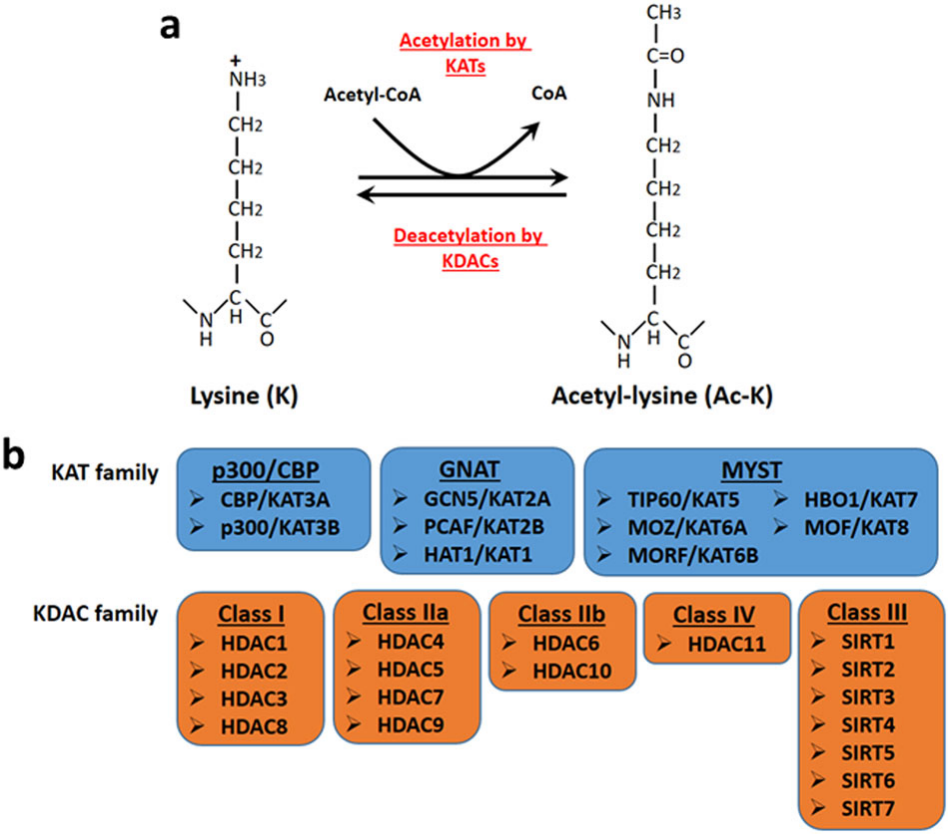
Regulation of lysine acetylation. **a** Lysine acetylation is a reversible posttranslational modification of proteins, including histones. Proteins can be acetylated at lysine residues (Ac-K) by specific enzymes, i.e., KATs, or deacetylated by KDACs. **b** Most canonical mammalian KATs are classified into three major families: The p300/CBP, GNAT and MYST family. KDACs are divided into two categories: classical Zn2+ -dependent HDACs and NAD+ -dependent sirtuin deacetylases. KDACs can be further grouped into class I, class IIa, class IIb, class III and class IV.

Lysine (K) acetylation depends on the use of Ac-CoA, and also nicotine adenine dinucleotide (NAD+) in the case of sirtuins, a class of lysine deacetylases (KDACs; HDACs), which use it as a co-substrate. This type of acetylation links metabolism with cell signaling, as Ac-CoA and NAD+ are key metabolites^18^, and modifies pathways that can be reversibly altered by deacetylases (Fig. 2). The K-acetylation and deacetylation of proteins were first studied in histones because of their roles in gene regulation. However, KATs and KDACs also acetylate nonhistone proteins in the nucleus or cytoplasm to regulate major biological processes^19^. Acetylation also occurs through nonenzymatic mechanisms and is affected by the availability of Ac-CoA^20,21^.

## KATs and KDACs and autophagy regulation

To date, approximately 40 mammalian proteins have been proposed to possess endogenous KAT activity. Thirteen are well characterized (canonical) and can be classified into three major families: the GCN5 (also known as KAT2A) and PCAF (also known as KAT2B) family (together members of the overarching GNAT family); the E1A binding protein p300 (encoded by EP300, also known as KAT3B) and CREB-binding protein (CBP, also known as KAT3A) family; and the MYST family, named for its founding members MOZ (also known as KAT6A), yeast Ybf2, Sas2, and Tip6 (also known as KAT5)^22^ (Fig. 2). All canonical KATs are predominantly localized in the nucleus and acetylate histones and nonhistone proteins. However, some KATs, such as p300, are nuclear but can be exported to the cytoplasm depending on intracellular signaling^23^. The substrate specificities of KATs are thought to be defined by their specific subcellular localization, their interacting proteins and the accessibility of lysine residues in substrate proteins^19^. KATs are found in unique complexes that influence their target specificities and their abilities to interact with other proteins^22^. More than 2,000 acetylation targets in the nucleus, cytoplasm, mitochondria and endoplasmic reticulum have been identified in human cells^24^.

The human genome encodes 18 KDACs, and they are divided into two major categories: zinc-dependent KDACs and NAD + -dependent sirtuin deacetylases (Table 1). On the basis of phylogenetic conservation and sequence similarities, zinc-dependent KDACs are further divided into four classes: class I, class IIa, class IIb and class IV^25^. Class I and class IV KDACs are localized in the nucleus, class IIb KDACs are cytoplasmic, and class IIa KDACs are primarily localized in the nucleus but are also found in the cytoplasm. Sirtuin (SIRT) deacetylases localize to different cellular compartments^26^, including the nucleus (SIRT1, SIRT6 and SIRT7), cytoplasm (SIRT2) and mitochondria (SIRT3, SIRT4 and SIRT5).

**Table 1:** KATs and KDACs on regulation of autophagy.

	Class	Representatives	Effect on autophagy	Main subcellular location	Ref.
KATs	p300/CBP family	CBP/KAT3A	Induction	Nucleus	^27^
			Inhibition	Nucleus	^30^
		p300/KAT3B	Inhibition	Nucleus	^28–30^
	GNAT family	GCN5/KAT2A	Inhibition	Nucleus	^31^
		PCAF/KAT2B	Induction	Nucleus	^32^
		HAT1/KAT1	Not reported	Nucleus	—
	MYST family	TIP60/KAT5	Induction	Nucleus	^33^
		MOZ/KAT6A	Not reported	Nucleus	—
		MORF/KAT6B	Not reported	Nucleus	—
		HBO1/KAT7	Not reported	Nucleus	—
		MOF/KAT8	Inhibition	Nucleus	^34^
KDACs	Class I	HDAC1	No effect	Nucleus	^37,38^
			Inhibition		^35,36^
		HDAC2	Induction	Nucleus	^37^
		HDAC3	Inhibition	Nucleus	^27^
		HDAC8	Inhibition	Nucleus	^42^
	Class IIa	HDAC4	Inhibition	Nucleus	^39,40^
		HDAC5	Inhibition	Nucleus	^40^
		HDAC7	Inhibition	Nucleus	^41^
		HDAC9	Inhibition	Nucleus	^43^
	Class IIb	HDAC6	Induction	Cytoplasm	^44–47^
		HDAC10	Induction	Cytoplasm	^48^
	Class IV	HDAC11	Not reported	Nucleus	—
	Class III (Sirtuins)	SIRT1	Induction	Nucleus	^49,50^
		SIRT2	Inhibition	Cytoplasm	^51^
		SIRT3	Induction	Mitochondria	^52^
		SIRT4	Induction	Mitochondria	^55^
		SIRT5	Induction	Mitochondria	^53^
		SIRT6	Induction	Nucleus	^54^
		SIRT7	Induction	Cytoplasm	^56^

In mammalian cells, KATs and KDACs play pivotal roles in autophagy regulation at multiple steps^27^ (Table 1). As protein acetylation is a major regulator of gene transcription, the epigenetic regulation of autophagy genes by KATs or KDACs may be important for autophagy regulation. Depending on the target proteins of KATs and KDACs, acetylation has the potential to induce or inhibit autophagy (Table 1).

### Regulation of autophagy by p300-dependent acetylation

Among KATs, p300 appears to acetylate many ATG proteins that regulate autophagy at multiple steps^27–29^ (Table 1). p300 depletion or specific p300 inhibitors can induce autophagy, whereas the overexpression of p300 inhibits autophagy^28,29^.

**Fig. 3:**
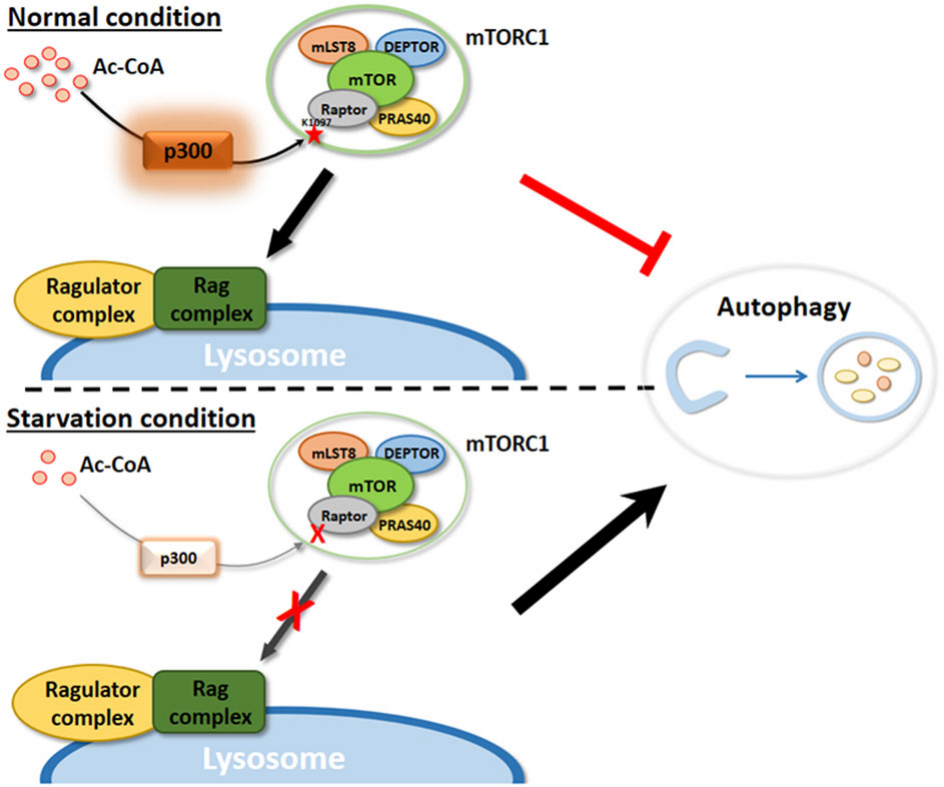
Regulation of autophagy by p300-dependent acetylation. Ac-CoA activates p300, which acetylates Raptor, leading to mTORC1 activation, which inhibits autophagy. Under nutrient (e.g., leucine) deprivation conditions, autophagy activation is mainly mediated by decreased Raptor acetylation to inhibit mTORC1.

Recently, we reported that under nutrient-depleted conditions, such as amino acid (AA) or leucine starvation, p300-dependent acetylation regulates autophagy through the acetylation of the mTORC1 component Raptor at K1097^30^. This acetylation of Raptor enables the interaction of mTORC1 with the Rag complex on the lysosomal membrane, where mTORC1 is activated. In this way, Raptor acetylation is mediated by leucine, and p300 activation results in mTORC1 activation and autophagy repression. Cells expressing an acetylation-dead mutant of Raptor (Raptor K1097R; KR) manifested autophagy activation without altered acetylation of autophagy-related proteins. Furthermore, p300 activation had no discernible effects on autophagy levels in Raptor KR-expressing cells or in cells where mTORC1 was inhibited. Thus, our data suggest that p300 activity (and leucine) inhibits autophagy primarily by activating mTORC1 rather than by altering the acetylation of other proteins^30^. This Ac-CoA-p300-Raptor regulation of autophagy via mTORC1 occurs in most cell types, including neurons (Fig. 3).

### The regulation of autophagy by other KATs

1. GNAT family
  In mammalian cells and Drosophila, GCN5 inhibits the biogenesis of autophagosomes and lysosomes by regulating the acetylation of TFEB at lysine 274 and lysine 279. The acetylation of TFEB disturbs its dimerization and its subsequent binding to target gene promoters, many of which regulate autophagy or lysosomal biogenesis^31^. PCAF, another member of the GNAT family, is reported to regulate autophagy through the inhibition of the mTORC1 pathway in some cancers, such as hepatocellular carcinoma^32^.
2. MYST family
  The MYST acetyltransferase family also appears to regulate autophagy. Serum deprivation leads to the association of the protein kinase GSK3β with TIP60/KAT5 and subsequent phosphorylation at serine 86 of TIP60. Phosphorylated TIP60 acetylates and activates ULK1^33^, which is essential for serum deprivation-induced autophagy. Additionally, under nutrient starvation, the induction of autophagy is coupled to a reduction in histone H4 lysine 16 (H4K16) acetylation through the downregulation of MOF/KAT8^34^.

### The regulation of autophagy by KDACs

1. KDACs family
  KDAC family members are important for the regulation of autophagy at several levels. HDAC1 has been reported to be overexpressed in hepatocellular carcinoma, and inhibition of HDAC1 induces autophagy to repress tumor cell growth^35^. Chemical or genetic HDAC1 inhibition also induces autophagy and lysosomal activity in HeLa cells^36^. However, knocking down HDAC2, but not HDAC1, inhibited autophagy in cardiomyocytes^37^. In contrast, the deletion of both HDAC1 and HDAC2 in mice blocked autophagic flux in skeletal muscle^38^. Knocking down class IIa HDAC4 leads to autophagy induction by increasing the expression levels of ATG proteins, including Beclin 1 and ATG7^39^.
  MicroRNA-dependent protein acetylation can also regulate autophagy. The regulation of HDAC4 and HDAC5 by miRNA-9 increased total LC3B and Rab7 levels^40^. Another study showed that the HDAC inhibitor suberoylanilide hydroxamic acid (SAHA) decreased HDAC7 protein levels in endometrial stromal sarcoma cells, producing an accumulation of autophagic vacuoles^41^. Recently, the HDAC8-selective inhibitor HMC was shown to induce autophagy in MCF-7 cells^42^. In response to hypoxia in a myoblast cell line, class IIa HDAC9 was significantly increased, thereby inhibiting intracellular autophagy through direct binding to the promoter regions of Beclin 1, ATG7 and LC3^43^.
  Class IIb HDAC6 is the only mammalian deacetylase that contains a ubiquitin-binding domain; therefore, when the ubiquitin-proteasome system is impaired, this HDAC has an important role in autophagy-dependent protein degradation^44^. Furthermore, HDAC6 overexpression increases autophagosome formation in various liver cancer cells by activating c‐Jun NH2‐terminal kinase (JNK)^45^. Additionally, HDAC6 depletion impairs serum starvation-induced autophagy. In serum-starved cervical carcinoma cells, increased LC3 acetylation resulting from HDAC6 inhibition correlated with decreased autophagic flux^46^. HDAC6 is also important for autophagosome-lysosome fusion. HDAC6 knockout impaired the fusion of autophagosomes and lysosomes by perturbing the formation of F-actin networks mediated by acetylation of cortactin^47^. Another class IIb deacetylase, HDAC10, promotes autophagy in neuroblastoma cells, and its knockdown disrupts autophagic flux^48^. In most studies, depletion of class I and IIa HDACs is associated with the enhanced expression of autophagy regulators involved in the induction steps, which results in the upregulation of autophagy. By contrast, inhibition of class IIb HDACs, such as HDAC6 and HDAC10, is more associated with the blockade of autophagic flux.
2. Sirtuins
  The sirtuin family of class III HDACs are NAD + -dependent deacetylases that modulate a variety of cellular processes, including energy metabolism, stress responses, cell survival and proliferation. The deacetylation reactions catalyzed by sirtuins are coupled to the cleavage of NAD + into nicotinamide and 1-O-acetyl-ADP ribose. Therefore, sirtuin activities are dependent on the availability of cellular NAD + and are influenced by cellular metabolic status. Seven sirtuins (SIRT1 to SIRT7) have been identified in the human genome, and recent studies have proposed important roles for all sirtuins in the regulation of autophagy^49–56^. In particular, SIRT1 deacetylates ATG5, ATG7 and LC3 and appears to positively regulate autophagy at several steps^49^.

## KATs and KDACs in neurodegenerative disease

Most of the neurodegenerative diseases in humans are caused by toxic intracytoplasmic, aggregate-prone proteins. Alzheimer’s disease (AD) pathology is characterized by amyloid-beta, an extracellular product derived from amyloid precursor protein (APP), and intracellular aggregated tau^57^. Parkinson’s disease (PD) is associated with the accumulation of alpha-synuclein (α-syn), and excess levels of this protein are sufficient to cause disease^58^. Huntington’s disease is a monogenic autosomal dominant disease caused by polyglutamine tract expansions in the huntingtin protein, while amyotrophic lateral sclerosis (ALS) can be either monogenic or complex. The monogenic causes of ALS include mutations in SOD1, FUS and TDP-43^59^. Importantly, all of these disease-causing intracytoplasmic proteins are autophagy substrates, and autophagy-upregulating drugs and genes enhance the clearance of these proteins and attenuate their toxicities in a range of animal models (flies, zebrafish and mice)^60–69^. Autophagy may also protect against neurodegeneration by dampening inflammatory-type processes and apoptosis^70,71^.

The importance of acetylation regulated by KATs and KDACs in neurodegenerative diseases has been highlighted by observations that imbalanced acetylation causes progressive neuron-specific loss, impaired neuronal function, and eventual neuronal death^72^. Many studies have reported that abnormal acetylation and deacetylation are linked to the pathogenesis of a variety of neurodegenerative diseases^73^ (Table 2). We briefly review the relationships between acetylation and different neurodegenerative diseases to reveal some of the complexities that may emerge when perturbing relevant modifying enzymes, as these may impact not only autophagy but also numerous other cellular processes pertinent to neurodegeneration.

1. Alzheimer’s disease (AD)
  p300-mediated histone H3 acetylation at the presenilin 1 (PS1) and beta-site amyloid precursor protein-cleaving enzyme 1 (BACE1) promoters is upregulated, consequently enhancing the expression levels of these genes in an AD model cell line^74^. Interestingly, p300 levels are significantly increased in an AD model cell line, suggesting that p300 regulates the expression of AD-related genes by controlling acetylation or their promoters. The overexpression of CBP leads to recovered loss of learning and memory in AD triple transgenic mice^75^. On the other hand, acetylation of tau can be modulated by p300 and SIRT1, and excess acetylated tau may contribute to tau-mediated neurodegeneration^76^. Interestingly, hyperactivation of p300/CBP activity has been reported to disrupt autophagic flux and cause excessive tau secretion^77^. PCAF knockout mice are resistant to Aβ-induced toxicity and memory deficits, an effect that has been attributed to the upregulation of the activity of the Aβ-degrading enzyme Neprilysin^78^. Impaired function of TIP60 has been described in the human AD hippocampus, and imbalanced TIP60/HDAC2 activity is observed in the brain of an APP *Drosophila* AD model, suppressing the activities of neuroplasticity genes, which can be rescued by overexpression of TIP60^79^.
  Inactivation of HDAC1 activity by the p25/Cdk5 complex, which is involved in neurodegenerative diseases, including AD, causes double-strand DNA breaks and neurotoxicity, which can be restored by HDAC1 overexpression^80^. Moreover, HDAC3 promotes tauopathy, whereas suppression of HDAC3 may affect not only nonamyloidogenic APP processing but also neuroprotective gene expression in vitro and in an AD mouse model^81^. Nuclear translocation of class II HDACs such as HDAC4 and HDAC6 is regulated by Aβ oligomers and the apolipoprotein E ε4 allele (apoE4), which is a critical AD risk factor, resulting in the downregulation of BDNF expression, which is important for controlling synaptic repair and synaptic plasticity^82^. HDAC6 binds to tau in the perinuclear aggresomal compartment, and HDAC6 levels are upregulated in the hippocampus of AD patients and AD mice^83^. By contrast, loss of HDAC6 improves learning and memory, α-tubulin acetylation and cognitive function in an AD mouse model^84^. In the brains of an AD mouse model, overexpression of SIRT1 inhibits Aβ oligomers and plaque burden and ameliorates behavioral deficits, suggesting a neuroprotective role for SIRT1 in AD^85^. Loss of SIRT2 induces microtubule stabilization and initiation of the subsequent autophagic-lysosomal pathway to degrade toxic Aβ oligomers in an AD-derived cell model^85^.
2. Parkinson’s disease (PD)
  While α-syn is believed to be an important effector of PD because of its activities in the cytoplasm, α-syn may also mediate neurotoxicity by interacting with histone H3, thereby inhibiting histone acetylation by inactivating several KATs, including CBP, p300 and PCAF^86^. The suppression of SIRT2 by either siRNA or a potent inhibitor prevents α-syn-dependent neurotoxicity, as well as the formation of α-syn inclusions in vitro and in a *Drosophila* model of PD^87^. However, the relationship between SIRT2 inhibition and α-syn aggregation is still unclear. In cortical Lewy bodies, α-syn colocalizes with the microtubule-binding proteins MABP1 and tau. Increased acetylation of α-tubulin by the inhibition of SIRT2 may promote the formation of α-syn aggregates by binding to microtubules, suggesting that stabilized microtubules can play an important role in neuroprotection^87^. Furthermore, the upregulation of SIRT2 prevents microtubule hyperacetylation and axonal degeneration^88^. The overexpression of SIRT1 increases the lifespan of an α-syn A53T PD mouse model and prevents the formation of α-syn aggregates. SIRT1 deacetylates HSF1 (heat shock factor 1) and thereby increases Hsp70 levels, suggesting that Hsp70 activation can inhibit the α-syn-mediated neurotoxicity of Hsp70^89^.
3. Huntington’s disease (HD)
  CBP is observed in the aggregates formed by mutant huntingtin (mHtt)^90^. PolyQ expansions, the mutations in the huntingtin protein, directly interact with and sequester CBP and PCAF in animal models, leading to transcriptional dysregulation^91^. In addition, loss of CBP from the nucleus impairs HAT activity and CBP-mediated gene expression, resulting in neuronal dysfunction and neuronal death^92,93^. Furthermore, soluble mHtt may enhance ubiquitination to accelerate CBP degradation via the ubiquitin-proteasome system^94^. mHtt acetylation at lysine 444 (K444) by CBP activation or HDAC1 inhibition promotes its trafficking to autophagosomes and subsequent clearance in primary neurons and a *C. elegans* HD model, suggesting a role in neuroprotection^95^. Similarly, HDAC6-mediated retrograde transport on microtubules may facilitate mHtt degradation through autophagy^44^. Several studies have reported that SIRT1 activity ameliorates mHtt-mediated toxicity in both cellular and animal models. In addition, mHtt suppresses SIRT1 deacetylase activity through a direct interaction causing SIRT1 to remain hyperacetylated, leading to the attenuation of SIRT1-regulated neuroprotective effects^96^. However, SIRT2 controls HD-related metabolism, such as cholesterol biosynthesis, leading to increased production of cholesterol, further increasing mHtt aggregation^97^.
4. Amyotrophic lateral sclerosis (ALS)
  Transgenic mice expressing the disease-causing mutant protein SOD1 G86D have low levels of histone H3 acetylation and CBP in motor neurons^98^. Similar to SOD ALS mouse models, low levels of CBP are found in the motor neurons of sporadic ALS patients^99^. Furthermore, SOD1 mutants may cause disrupted axonal transport and contribute to the loss of mitochondria from axons because of defective microtubule-dependent trafficking^100^. Interestingly, decreased acetylation of α-tubulin is observed in HAT Elp3-deficient cortical neurons^101^. Knocking out HDAC6 in SOD1 G93A-expressing mice reduces motor neuron degeneration and increases acetylated α-tubulin without affecting disease onset^102^. However, conflicting functions of HDAC6 in mice harboring mutant SOD1 have also been reported. Inhibition of HDAC6 promotes the formation of large mutant SOD1 aggregates, which is accompanied by the increased acetylation of α-tubulin and enhanced microtubule retrograde transport. Interestingly, HDAC6 specifically binds to mutant SOD1 through SOD1 mutant interaction region (SMIR) motifs^103^. Other ALS-causing proteins, namely, TDP-43 and FUS/TLS, appear to interact with HDAC6 to control mRNA expression levels. Moreover, the downregulation of TDP-43 lowers the levels of HDAC6, leading to disrupted aggregate formation^104^. G93A SOD1 induces DNA damage and subsequently facilitates apoptosis by activating p53^105^. p53 K320 acetylation is modulated by p300/CBP and PCAF and produces neuroprotective effects, including neurite outgrowth and axon regeneration^106^. Furthermore, p53 K382 acetylation is controlled by p300/CBP and SIRT1, thereby facilitating neuronal apoptosis^106^.

**Table 2:** KAT and KDAC enzymes and neurodegenerative diseases.

Disease		Class	Prognostic relevance	Molecular evidence	Molecular consequence	Opposite effect	Ref.
AD	KAT	p300	Upregulated in N2a/APPswe cells	Binding to the PS1 and BACE1 promoters and acetylation	p300 increases PS1 and BACE1 expression levels and increases the expression of Aβ.	Overexpression of p300 induces neuronal cell death linked to AD pathology.	^74^
			Increased p-p300 (Ser1834) in CA1 of AD brain	p-p300-positive neurons colocalize with p-tau	p300 leads to tauopathy.		^107^
			Decreased in AD brain		Aβ induces posttranslational degradation of p300.		^108^
		CBP	Decreased in AD brain			Overexpression of CBP rescues learning and memory loss in AD mice.	^75,108^
		PCAF	Decreased in AD brain	Regulation of NEP and low level of SRIH		Knocking out PCAF reduces Aβ-mediated toxicity.	^78,108^
		TIP60	Human AD hippocampus, APP Drosophila	Suppression of synaptic plasticity gene	TIP60 suppresses synaptic plasticity.		^79^
			Drosophila CNS	Binding to APP through AICD	Tip60 functions neuroprotection.		^109^
	KDAC	HDAC1	Decreased in frontal cortex and hippocampal region of AD patients	Inactivation by the p25/Cdk5 complex	Inactivated HDAC1 causes dsDNA breaks and neurotoxicity.	Overexpression of HDAC1 leads to neuroprotection.	^80,108^
		HDAC2	Upregulated in AD brain	Inhibition of neuronal gene expression	HDAC2 causes disruption of synaptic plasticity and neuronal development.	HDAC2 deficiency improves the increased synapse numbers and memory.	^110^
		HDAC3	In vitro and AD mice model		HDAC triggers not only tau phosphorylation but also Aβ metabolism in AD cellular and animal models.	Inhibition of HDAC3 decrease tau phosphorylation and Aβproduction, leading to a neuroprotective effect.	^81^
		HDAC4/HDAC6	Increased nuclear localization	Nuclear localization by Aβ oligomers and ApoE4	HDAC4/6 affect inhibition of BDNF expression, which controls synaptic repair and plasticity.		^82^
		HDAC6	Upregulated in AD patient, mice model and during AD progression	Binding to tau in perinuclear aggresome, deacetylation α-tubulin and tau.	HDAC6 leads to tauopathy.	Knocking down HDAC6 improves learning and enhanced memory in AD mice.	^83,84,111^
		SIRT1	Decreased in AD brain	Binding to tau	Loss of SIRT1 promotes p-tau and consequent tauopathy.	Overexpression of SIRT1 prevents tauopathy.	^112^
					Reduced level of SIRT1 induces Aβ oligomers.	Upregulation of SIRT1 suppresses Aβ production through control of γ-secretase activity.	^85,112^
		SIRT2	AD-derived cell model	Inhibition of microtubule stabilization		Loss of SIRT2 induces microtubule stabilization and degrades toxic Aβ oligomers by autophagy.	^85^
HD	KAT	CBP	Decreased in the hippocampus of mutant HdhQ7/Q111 mice		Reduced level of CBP impairs memory and induces neuron death.		^113^
			Low expression of CBP in HD	Binding to polyQ expansions	Low level of CBP reduces acetyltransferase activity, leading to mHtt accumulation and impaired intracellular trafficking.	CBP overexpression promotes increased acetylation and degradation of mutant Htt.	^95^
			Loss of CBP in HD Drosophila model and primary neurons	Acetylation of mHtt at K444	CBP-mediated mHtt acetylation at K444 triggers trafficking to autophagosome for mHtt degradation by autophagy.		^95^
		PCAF	Loss of PCAF in HD Drosophila model	Binding to mHtt	Reduced PCAF level triggers neurodegeneration.		^114^
	KDAC	HDAC6	SBMA Drosophila model		HDAC6 regulates mHtt degradation via autophagy.		^115^
		SIRT1		Controls BDNF expression and mTORC1- CREB-regulated transcription	SIRT1 inhibits mHTT–mediated interference with mTORC1 activity and improves mHtt toxicity.		^116^
		SIRT2			SIRT2 inhibition offers neuroprotection by decreasing sterol biosynthesis.		^117^
PD	KAT	CBP, p300, PCAF				Inhibition of CBP, p300 and PCAF induce histone H3 deacetylation	^86^
	KDAC	SIRT1	Downregulated in PD brain	Extension of the life span of αSyn A53T-expressing mice	SIRT1 prevents the formation of αSyn aggregates.		^89^
				Deacetylation of HSF1 and increase in Hsp70	SIRT1 Inhibits αSyn-mediated neurotoxicity by activating molecular chaperones.		^89^
		SIRT2	Upregulated in PD	Binding to α-tubulin	SIRT2 inhibits α-tubulin hyperacetylation and leads to axonal degeneration.	Inhibition of SIRT2 promotes αSyn mediated neurotoxicity through α tubulin acetylation.	^87,88^
ALS	KAT	CBP	Low level of CBP in motor neuron of ALS patients				^99^
		P300, CBP, PCAF		Acetylation of p53 K320	Neuroprotection		^106^
		P300, CBP		Acetylation of p53 K382	Neuronal apoptosis		^106^
	KDAC	HDAC2	Upregulated mRNA				^118^
		HDAC11	Downregulated mRNA				^118^
		HDAC6	Downregulated in TDP-43 and FUS/ALS	Binding to SOD mutants	Disrupted aggregation formation	Knockout of HDAC6 prevents motor neuron degeneration in SOD G93A mice.	^102,119^

## Concluding remarks

Although the mechanisms remain incompletely understood, accumulating evidence indicates that different KATs and KDACs play pivotal roles in autophagy regulation at multiple steps of the pathway. New links between protein acetylation and autophagy control are likely to emerge. Acetylation also plays a crucial regulatory role in pathological conditions, particularly in neurodegenerative diseases and cancer. Thus, identifying how acetylation impacts various processes involved in neurodegenerative diseases, including autophagy, will help to inform suitable therapeutic strategies.
